# A knowledge graph to interpret clinical proteomics data

**DOI:** 10.1038/s41587-021-01145-6

**Published:** 2022-01-31

**Authors:** Alberto Santos, Ana R. Colaço, Annelaura B. Nielsen, Lili Niu, Maximilian Strauss, Philipp E. Geyer, Fabian Coscia, Nicolai J. Wewer Albrechtsen, Filip Mundt, Lars Juhl Jensen, Matthias Mann

**Affiliations:** 1 NNF Center for Protein Research, Faculty of Health Sciences, University of Copenhagen, Copenhagen, Denmark; 2 Li-Ka Shing Big Data Institute, University of Oxford, Oxford, UK; 3 Center for Health Data Science, Faculty of Health Sciences, University of Copenhagen, Copenhagen, Denmark; 4 OmicEra Diagnostics GmbH, Planegg, Germany; 5 Department of Proteomics and Signal Transduction, Max Planck Institute of Biochemistry, Martinsried, Germany; 6 Department of Biomedical Sciences, Faculty of Health and Medical Sciences, University of Copenhagen, Copenhagen, Denmark; 7 Department for Clinical Biochemistry, Rigshospitalet, University of Copenhagen, Copenhagen, Denmark

**Keywords:** Biomarkers, Software, Proteomics, Data integration, Data mining

## Abstract

Implementing precision medicine hinges on the integration of omics data, such as proteomics, into the clinical decision-making process, but the quantity and diversity of biomedical data, and the spread of clinically relevant knowledge across multiple biomedical databases and publications, pose a challenge to data integration. Here we present the Clinical Knowledge Graph (CKG), an open-source platform currently comprising close to 20 million nodes and 220 million relationships that represent relevant experimental data, public databases and literature. The graph structure provides a flexible data model that is easily extendable to new nodes and relationships as new databases become available. The CKG incorporates statistical and machine learning algorithms that accelerate the analysis and interpretation of typical proteomics workflows. Using a set of proof-of-concept biomarker studies, we show how the CKG might augment and enrich proteomics data and help inform clinical decision-making.

## Abstract (web summary)

A knowledge graph platform integrates proteomics with other omics data and biomedical databases.

## Main

The paradigm of evidence-based precision medicine has evolved toward a more comprehensive analysis of disease phenotypes. This requires seamless integration of diverse data, such as clinical, laboratory, imaging and multiomics data (genomics, transcriptomics, proteomics or metabolomics)^1^. Recently, we found that a more fine-grained definition of disease that combines clinical and molecular data can provide a deeper understanding of individuals’ disease phenotypes and reveal candidate markers of prognosis and/or treatment^2–4^. Moreover, multiomics data can generate new hypotheses that ultimately translate into clinically actionable results^5^. The biomedical research community has long recognized the need to collect, organize and structure the relevant data, resulting in community-wide adoption of multiple biomedical databases (Supplementary Table 1). However, harmonization and integration is still challenging because it is often diverse, heterogeneous and distributed across multiple platforms. Moreover, much scientific data and knowledge are only ‘stored’ within millions of unstandardized journal publications.

Over the last decade, mass spectrometry (MS)-based proteomics has advanced greatly and now provides an increasingly comprehensive view of biological processes, cellular signaling events and protein interplay^6^. However, currently used MS-based proteomics workflows were conceptualized more than a decade ago, and rapidly increasing data volumes are posing new challenges for the field. An even larger and growing bottleneck in high-throughput proteomics is the difficulty of interpreting the quantitative results to formulate biological or clinical hypotheses. Only a handful of tools have been aimed at alleviating this problem^7,8^ There is a need for solutions that integrate multiple data types while capturing the relationships between the molecular entities and the resulting disease phenotype. Moreover, we see an increasing need for more inclusive solutions that provide those with little expertise with tools for extracting high-quality information from proteomics data in a more user-friendly manner. Therefore, a knowledge-based platform that integrates a range of databases and scientific literature information with omics data into an easy-to-use workflow would empower discovery science and clinical practice.

Networks and graphs have emerged as natural ways of representing connected data, including also in biology^9–11^. Efforts during the last decade have organized large amounts of diverse information as collections of nodes (entities) and edges (relationships)^12–16^. The resulting flexible structure, called a knowledge graph, quickly adapts to complex data with their relationships and enables the efficient use of network analysis techniques to identify hidden patterns and knowledge^13,17–19^.

Here we take this concept into a new direction and describe a knowledge graph framework that facilitates harmonization of proteomics with other omics data while integrating the relevant biomedical databases and text extracted from scientific publications. Termed the CKG, it constitutes a graph database of millions of nodes and relationships. It allows clinically meaningful queries and advanced statistical analyses, enabling automated data analysis, knowledge mining and visualization. The CKG incorporates community efforts by building on scientific Python libraries^20^, which also makes the platform reliable, maintainable and continuously improving. The entire system is open source and permissively licensed (by the MIT license). It enables repeatable, reproducible and transparent analysis in both standard workflows and interactive exploration based on Jupyter notebooks.

## Results

### Overview of CKG architecture

**Fig. 1:**
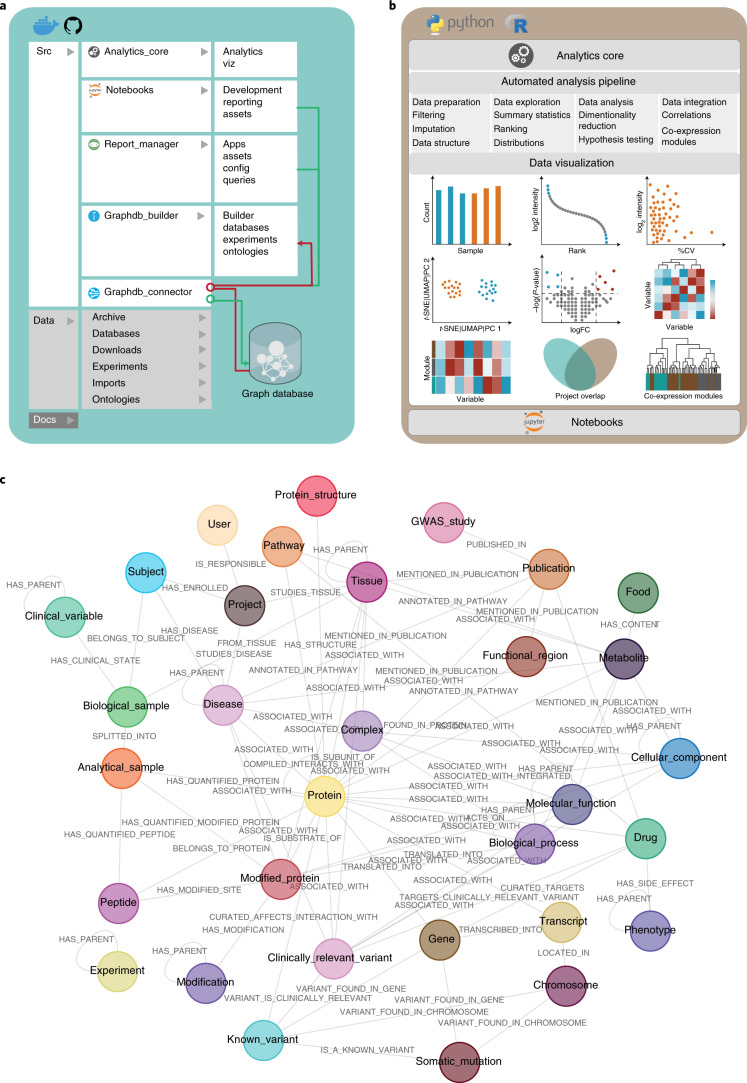
The clinical knowledge graph architecture. **a**, The CKG architecture is implemented in Python and contains several independent modules responsible for connecting to the graph database (graphdb_connector), building the graph (graphdb_builder), analyzing and visualizing experimental data (analytics_core), displaying and launching multiple applications (report_manager); it also contains a repository of Jupyter notebooks with analysis examples (notebooks). The code is accessible at https://github.com/MannLabs/CKG or as a complete Docker container. **b**, The CKG analytics core implements multiple up-to-date data science algorithms for statistical analysis and visualization of proteomics data: data preparation, exploration, analysis and visualization. This library can also be used directly within Jupyter notebooks, independently of the other CKG modules, and to analyze other omics types. **c**, The CKG graph database data model was designed to integrate multi-level clinical proteomics experiments and to annotate them with biomedical data. It defines different nodes (for example, Protein, Metabolite and Disease) and the types of relationship connecting them (for example, HAS_PARENT and HAS_QUANTIFIED_PROTEIN). FC, fold change; Src, source code.

The CKG includes several independent functional modules that (1) format and analyze proteomics data (analytics_core); (2) construct a graph database by integrating available data from a range of publicly accessible databases, user-conducted experiments, existing ontologies and scientific publications (graphdb_builder); (3) connect and query this graph database (graphdb_connector); and (4) facilitate data visualization, repository and analysis via online reports (report_manager) and Jupyter notebooks (Fig. 1a,b). This architecture seamlessly harmonizes and integrates data as well as user-supplied analysis. It also facilitates data sharing and visualization as well as interpretation based on detailed statistical reports annotated with biomedical knowledge, generating clinically relevant results. In the next several sections, we describe individual modules and the knowledge graph construction process.

### The analytics core as an open proteomics analysis framework

The first step in downstream analysis of proteomics data requires a comprehensive and versatile collection of statistical, machine learning and visualization methods. MSstats and Perseus have advanced proteomics by providing multi-purpose statistical and bioinformatics tools for the analysis of quantitative MS-based proteomics data^7,8^. Taking these and other efforts as a reference, we developed the analytics core of CKG that encompasses the desired functionality in a transparent and efficient manner. We chose Python and its associated scientific stack because this allows us to adopt well-tested and up-to-date algorithms while avoiding reimplementing already existing methods. The functionality implemented in the analytics core centers on statistical and visual data representation and covers all main computational areas, such as expression, interaction and post-translational, modification-based proteomics (Fig. 1b).

We designed the analytics core to comprise the main steps in a data science pipeline: data preparation (filtering, normalization, imputation and data formating), data exploration (summary statistics, ranking and distributions), data analysis (dimensionality reduction, hypothesis testing and correlations) and visualization. The analytics core goes beyond previous efforts by integrating the analysis of other data types in addition to proteomics (that is, clinical data, multi-omics, biological context and text mining). Furthermore, to complement the extensive Python portfolio, we incorporated functions optimized in the R language (that is, SAMR and WGCNA^21–23^) (Supplementary Table 2).

We included a visualization module (viz) that covers both basic plots (for example, bar plot or scatter plot) and more complex ones (for example, network, Sankey or polar plots) using the graphing library Plot.ly (https://plot.ly/), a graphing library compatible with Python and R. This way, visualizations created by the CKG framework can be easily exported and used from other languages (Methods).

Owing to its modular design, the analytics core can be used within the CKG framework but also independently by importing it from Python. Similarly, the analysis and visualization functionalities are not limited to proteomics data but can handle any type of data in matrix format. The open design promotes easy integration of new analysis methods and visualizations. Our integration of Jupyter notebooks, an open-source tool that allows mixing of text, graphics, code and data in a single document^24–27^, enables standard or bespoke analysis pipelines, including addition of existing or user-implemented functionality from the Python or R ecosystems (Methods).

### Building and populating a graph database

To achieve seamless annotation and integration of proteomics data with other omics experiments and/or literature information, we constructed a graph database that naturally connects large and heterogeneous data (Supplementary Fig. 1a). We chose the open-source Neo4J database platform as our current backend because of its performance, industry acceptance and associated Cypher query language (https://neo4j.com/) (Supplementary Fig. 1b). To build the knowledge graph, we first wrote a library of parsers (graphdb_builder) with associated configurations for each ontology, database and type of experiment. These parsers download the data from online resources, extract information and generate entities (nodes) and relationships, both of which can have attributes, such as name or description, in protein nodes. The parsers use paired configuration files that specify how ontologies, databases or experiments need to be interpreted. This design allows unrestricted integration of new resources or processing tools. Their output formats tend to change from time to time, but this only affects one parser/configuration that can be easily adapted. For example, the current CKG’s proteomics parser accepts output data from data-dependent or data-independent acquisition modes from commonly used programs, such as MaxQuant, Spectronaut, FragPipe or DIA-NN^28–31^, or the community standard format mzTab for MS-based proteomics and metabolomics data^32^. The CKG can be easily extended to accept additional data outputs as new processing programs emerge.

Once the ontology, database and experiment files are standardized, formatted and imported, the graphdb_builder module loads them into the graph database with a set of Cypher queries that create the corresponding nodes and relationships (Methods). Our data model connects 36 different node labels with 47 different relationship types (Fig. 1c). To make experimental proteomics data^33^, we designed a data model capable of supporting storage of standardized metadata (such as studied disease and interventions) around each research project, defining unique identifiers for enrolled individuals, collected biological samples and analyzed samples (Supplementary Fig. 2a). It enables predefined queries about experimentally determined protein hits, regarding their association to the diseases studied (ontological associations), drugs or annotated Gene Ontology terms and pathways. These types of queries provide insights into altered functions, suggesting drugs for regulated proteins and connections to metabolites to reveal possible confounding factors.

### CKG includes millions of nodes and relationships

The CKG database is continuosly growing and currently collects annotations from 26 biomedical databases using ten ontologies and organizes this information into almost 20 million nodes connected by 220 million relationships (Supplementary Fig. 1a). More than 50 million of these relationships involve ‘Publication’ nodes linking scientific publications about studies in the human system, coded with PubMed identifiers, to proteins, drugs, diseases, functional regions and tissues (Supplementary Fig. 3a). They were derived from named-entity recognition on almost 7 million abstracts and full-text articles (8.5% of overall publications based on full text but 20.4% from the past 10 years)^34,35^, thus encapsulating aspects of the accumulated biomedical knowledge in peer-reviewed publications.

We found the graph structure to be easily scalable, facilitating the integration of new ontologies, databases and experiments. Furthermore, this inherent flexibility allows nodes and relationships originally designed to provide biomedical context primarily for large-scale proteomics data interpretation to be readily remodeled to integrate other omics datasets. For instance, to integrate metabolomics data, we can link metabolite nodes already present in the database to analytical samples. The existing relationships connecting metabolites to nodes, such as pathways, proteins, tissue or disease, aid interpretation and seamless integration with proteomics results.

The CKG framework provides an infrastructure that facilitates exploiting the existing connections in the graph and the already implemented and optimized graph algorithms in Neo4j and Python libraries, such as NetworkX^36^. For example, when a new project is integrated, the default analysis can identify similar projects in the graph, and the results of these comparisons are shown in the project report. This functionality compares projects based on the overlap of identified proteins (Jaccard and overlap similarity) or similar protein profiles (Pearson correlation). In addition, the CKG provides a framework for the application of network analysis and machine learning algorithms. Learning on the graph structure using graph representation learning^37,38^ can enhance the prediction of new links, a strategy known as link prediction or graph completion^39,40^ (Methods).

### A framework to extract actionable knowledge

**Fig. 2:**
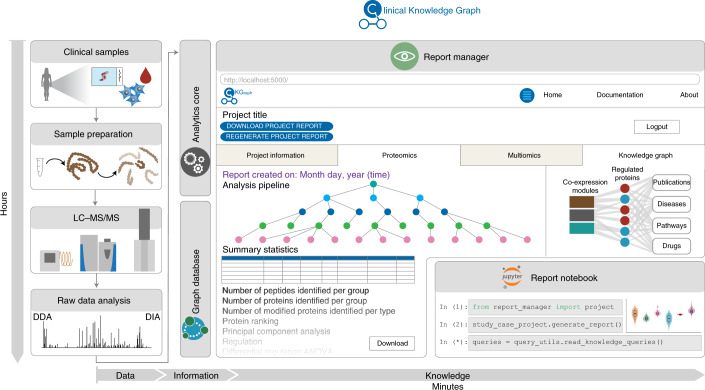
Automated statistical reports. The report_manager module includes a collection of dashboard applications that interface with the database and display statistics, create and upload new projects and report results from automated analysis pipelines. These reports include multiple tabs, one for each data type analyzed; a multiomics tab when multiple data types are analyzed together; and a knowledge graph that summarizes the results obtained in the previous tabs. This report can be viewed in the browser or accessed through a Jupyter notebook (Methods). LC–MS/MS, liquid chromatography with tandem mass spectrometry. DDA, data dependent acquisition; DIA, data independent acquisition.

A main goal of the CKG is to combine the power of the analytics module with the massive prior information integrated into the graph database to best interpret MS-based proteomics or other omics experiments. Harmonization of these heterogeneous but connected data sources enables standard analysis pipelines that report results automaticaly, replacing weeks of manual work in a more consistent format. These standard reports provide an initial evaluation of the quality of the generated data, highlight relevant hits and contextualize these hits in relation to different biomedical components in the graph. The report manager component (report_manager) orchestrates the creation and updates experimental projects and the automatic analysis, visualization and knowledge extraction (Fig. 2).

The report manager was implemented as a collection of dashboard applications that interface with the database for an overview of the knowledge graph (Home), to create and upload new clinical proteomics projects (Project Creation and Data Upload) and to run automated analysis pipelines (ProjectApp). This defines a workflow from project idea to knowledge-based analysis report (Supplementary Fig. 2b). The Project Creation and Data Upload steps generate the nodes and unique identifiers in the CKG for the project, the enrolled cohort individuals (if applicable), biological samples collected and analytical samples analyzed by MS-based proteomics. This also includes connections to diseases, tissues and clinical interventions (Supplementary Fig. 2c), which provide prior knowledge, such as known proteins associated with these diseases and tissues or literature related to them. Once the clinical and/or proteomics data are ready and processed, they are integrated into the graph by the ‘Data Upload’ dashboard app (Supplementary Fig. 2d).

Uploading the data triggers the builder’s import and load process and generates all the necessary relationships within the new project, including the links to proteins, peptides and protein modifications quantified in the project. The new links are used by the report manager module to analyze the different data types relevant for the project. These analyses are predefined using configuration files that break down the steps of the complete analysis workflow in a standardized, flexible and scalable manner. They describe the input data, the analysis to be performed and the parameters to be used as well as how the results are visualized. The final report is then the sequence of created visualizations for each data type divided into different tabs in the dashboard (plots and tables).

Apart from being viewable in the browser, all reports, analysis results and visualizations can be downloaded as a single compressed file containing tables and figures in ready-to-publish format. Moreover, they are also available in hierarchical data format (HDF5), a standard and scalable file format supported by many programming languages, enabling interoperability. This design facilitates continuous integration of newly developed analyses and visualizations (Supplementary Fig. 4). Furthermore, configuration files can be shared, which fosters transparency and facilitates replicability and reproducibility.

### Automated CKG analysis for liver disease biomarker discovery

**Fig. 3:**
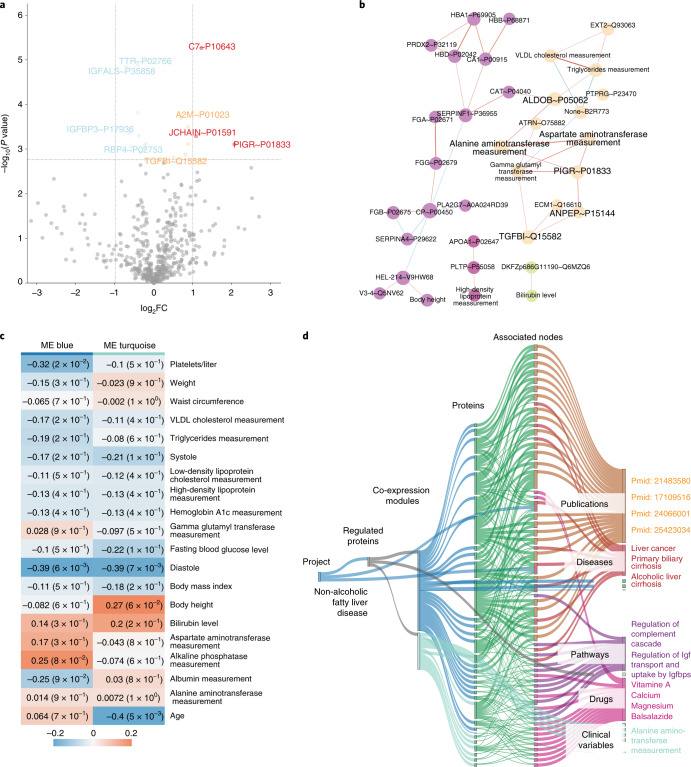
Default analysis of the nonalcoholic fatty liver disease study. The CKG’s automated analysis pipeline reproduced previous results (Niu et al.^41^). Visualizations were generated automatically by the report manager and downloaded from the dashboard app. **a**, Differential regulation. The volcano plot is part of the analysis performed on the proteomics data (Proteomics tab) and shows the dysregulation of proteins involved in immune system regulation and inflammation (for example, C7, JCHAIN, PIGR and A2M) (two-sided *t*-test comparison of cirrhosis versus healthy—BH FDR < 0.05) (upregulated: orange/red (fold change (FC) > 2); downregulated: light blue/blue (FC > 2)). **b**, Global clinical proteomics correlation analysis. The network finds correlations between proteins and quantitative clinical variables (Spearman correlation) and shows that clinical liver enzyme values cluster together with HbA1c, PIGR, TGFBI, ANPEP, C7 and other candidate biomarkers of liver fibrosis and cirrhosis (nodes colored by cluster—Louvain clustering). **c**, WGCNA. This analysis generates a heat map showing the association of co-expression modules with clinical variables (correlation and *P* value). This plot shows a higher positive correlation between the co-expression blue module and clinically measured liver enzyme levels in the plasma. **d**, Knowledge summary. This Sankey plot shows a summary of all the results obtained connecting co-expression modules to proteins, clinical variables, related diseases and pathways, drugs and publications. Betweenness centrality prioritizes the nodes to be visualized among all the associations found in the knowledge graph (top 15 central nodes for each node type). VLDL, very-low-density lipoprotein. ME, module eigengenes.

To show how CKG accelerates and extends both the analysis and interpretation of the data, we use its default pipeline on a proteomics study of nonalcoholic fatty liver disease (NAFLD)^41^ (Fig. 3).

For the clinical data, the CKG default analysis pipeline automatically summarized clinical characteristics of the cohort and highlighted variables with significant variances between the studied groups (healthy and normal glucose tolerance (NGT), type 2 diabetes (T2D), NGT with NAFLD and T2D with NAFLD and cirrhosis). This confirmed significant differences in the levels of the liver enzymes alanine aminotransferase, aspartate aminotransferase and alkaline phosphatase measurement or hemoglobin A1c (HbA1c), among others.

The proteomics default analysis started with an overview of the identified peptides and proteins as well as a detailed summary of descriptive statistics of the proteomics data matrix. The CKG continued with a visualization of proteome coverage, dynamic range, protein coefficients of variation (CVs) among samples and sample quality control based on known tissue quality markers^42^. These rankings were automatically annotated with curated information mined from the knowledge graph, in this case highlighting markers already used in the clinic linked to NAFLD or cirrhosis (Supplementary Fig. 5).

The default data analysis uses principal component analysis to reduce the dimensionality of features for an overview of the data. ANOVA with post hoc tests then determines statistically significant differences across all studied groups and between particular pairs of groups (post hoc analysis). Post hoc tests are presented as interactive volcano plots, with information about upregulated and downregulated proteins with a predefined significance threshold (that is, fold change > 2 and false discovery rate (FDR) < 0.05) (Fig. 3a). The CKG automatically reproduced our previous results showing dysregulation of proteins involved in immune system regulation and inflammation, such as C7, JCHAIN, PIGR and A2M, a known marker of liver fibrosis for which CKG reported 14 publications, confirming this connection. In addition, the CKG highlighted the TTR-RBP complex (TTR and RBP4) as downregulated in patients with cirrhosis compared to healthy individuals. This complex is involved in retinoid metabolism, whose dysregulation is linked to hepatic diseases and alteration of the extracellular matrix deposition, leading to fibrosis^43^. Furthermore, the report revealed literature and database associations among the regulatory role of CD5L and cirrhosis, hepatocellular carcinoma and other liver diseases^44^. These metabolically interesting findings were missed in our manual analyses but were prioritized by the CKG’s automated pipeline, which extracted significantly regulated proteins in the different conditions. To visualize correlated protein changes as a network, the default analysis connected proteins with significant associations (Pearson correlation coefficient > 0.5 and FDR < 0.05). Using the Louvain algorithm to detect clusters of highly correlating proteins^45^ revealed potentially clinically relevant connections, such as an association of a cluster composed by PIGR, and DPP4 and TGFBI with liver fibrosis. The background knowledge of millions of protein interactions enabled the CKG to identify six main clusters grouping extracellular matrix remodelers, complementary components and inflammation markers, which connected two of the candidate biomarkers (PIGR and JCHAIN).

Associating the differentially regulated proteins to drugs, diseases and publications and to enriched biological processes and pathways identified additional dysregulated pathways in NAFLD that were overlooked in our previous analysis. These included ‘regulation of insulin-like growth factor (IGF-1) transport’ and ‘uptake by insulin-like growth factor binding proteins (IGFBPs)’, which were linked to changes in IGFBP3 acid-labile subunit (IGFALS). Notably, such associations have recently been investigated for causal relationships and therapeutic potential in NAFLD^46,47^. The CKG also reported protein–disease associations, indicating possible shared disease mechanisms with liver cancer, hepatitis and pancreas disease.

The presence of various data types in the project triggered the default multi-omics analysis pipeline. In a global clinical proteomics correlation analysis^48^, clinical liver enzyme values clustered with HbA1c, fasting glucose levels and several candidate biomarkers of liver fibrosis and cirrhosis (such as PIGR, TGFBI, ANPEP and C7) (Fig. 3b). The CKG also used WGCNA to obtain modules of co-expressed proteins instead of individual proteins that are related to clinical variables (Fig. 3c).

Finally, the automated analysis pipeline summarized all clinical, proteomics and multi-omics analyses as a graph with all the regulated proteins and the relationships extracted from the knowledge graph (such as diseases, drugs, interactions and pathways) using betweenness centrality to prioritize and reduce the number of nodes presented (Fig. 3d).

The entire default pipeline took less than 5 min but captured basically all insights gleaned from our previous manual analysis, which had taken weeks. The interpretation of the differentially abundant proteins had comprised time-consuming literature and database searches for known/published protein–disease associations and knowledge gathering^41^, but the CKG revealed them to be to be incomplete.

### CKG enables multi-proteomics data integration for cancer biomarker discovery and validation

**Fig. 4:**
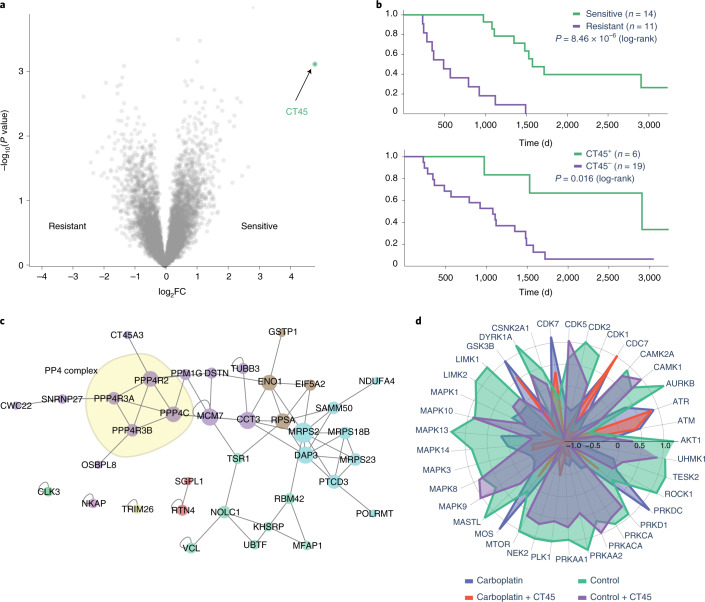
CKG analysis of multi-level clinical proteomics. **a**, The CKG highlights CT45 as the only protein significantly regulated when comparing ovarian tumor tissue from chemo-resistant and chemo-sensitive patients (*n* = 25; SAMR s0 = 2; BH FDR < 0.05) (data from Coscia et al.^3^). **b**, The CKG’s analysis pipeline estimates the survival function for the clinical groups sensitive and resistant (two-sided log-rank test) with corresponding high (top 25%) and low (remaining 75%) CT45 expression and confirms the significantly longer disease-free survival of the high-CT45-expression group. **c**, Interaction proteomics revealed subunits of the PP4 phosphatase complex as direct interactors of CT45, shown by the CKG as clusters in the PPI network, confirming known interactors and highlighting potential novel ones (nodes colored by cluster). **d**, Phosphoproteomic analysis in the CKG identified significantly regulated sites and linked them to upstream kinase regulators. Among these kinase regulators, CDK7, CDC7, ATR and ATM are highly affected by the action of carboplatin. FC, fold change.

To explore the multi-analysis capabilities of the CKG, we reanalyzed a recent study in which we identified cancer/testis antigen family 45 (CT45) as a biomarker for long-term survival in ovarian serus adenocarcinoma and described its mode of action^3^. Multi-dimensional proteomics, phosphoproteomics and interactomics were modeled as different connected projects in the CKG and analyzed independently using the default analysis adapted to each data type (proteomics, interactomics and phosphoproteomics). The CKG reproduced CT45 as significantly higher expressed in patients with long-term remission after chemotherapy (Fig. 4a,b). The CKG also confirmed that there was virtually no previous knowledge about cellular roles and functions of CT45 but produced 24 potential interactors of CT45, four of them belonging to the PP4 complex and contributed by a human interaction map^49^ (Fig. 4c).

The PP4 complex has been linked to DNA damage repair^50^, which, together with the fact that patients had undergone chemotherapy inducing DNA interstrand crosslinks, prompted us to investigate the phosphoproteome in cell line models^3^. The CKG’s default analyis of the signaling response of CT45-expressing cells versus controls indeed revealed activation of the relevant DNA damage pathways. The CKG pinpointed several known DNA damage kinases (that is, ATM/ATR) and their correponding regulated substrates (SMC1A_S966/S957, NBN_S615 and PBRM1_S948), providing deeper insights into the mechanisms of action of carboplatin and its negative regulation of proliferation through DNA damage repair. Additionally, the CKG exposed several other relevant kinases and associations that had eluded manual analysis, such as site-specific activation of MAPK activity as well as differences in CDC7 and CDK7 substrate regulation (Fig. 4d). Although not used in this example, the CKG includes similar capabilities for genomic and transcriptomics data, as well as other omics data types, for further integration.

### Using CKG to prioritize treatment options for chemorefractory cases

After standard treatment options have been exhausted in end-stage cancer, molecular profiling might still reveal druggable targets and opportunities for drug repurposing^51,52^, and we previously used proteomics profiling of cancer tissue to identify alternative targeted strategies^2,4^. To similar ends, CKG currently mines more than 350,000 connections between proteins and approved or investigational drugs targeting them (Supplementary Table 4).

**Fig. 5:**
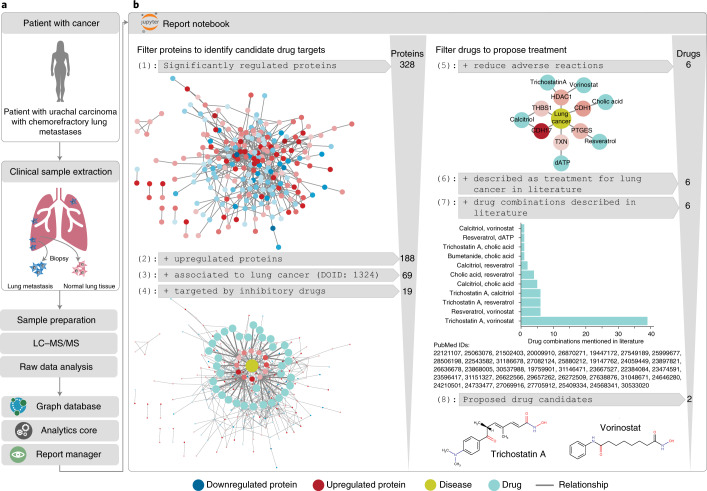
CKG helps to prioritize alternative treatments. **a**, Re-analysis of Doll et al.^2^ resulted in more than 300 significantly regulated proteins differentially expressed in uracal carcinoma, and the analysis was then extended to prioritize candidate drug targets and treatments. **b**, Simplified representation of the major steps included in the extended downstream analysis. The pipeline mined the CKG database to identify upregulated proteins known to be linked to the studied disease, found inhibitory drugs for these proteins, retrieved reported side effects and ultimately identified possible combinations of the prioritized drugs based on co-mentioning in scientific literature. A Jupyter notebook with the complete analysis pipeline to prioritize candidate treatments can be found in notebooks/reporting, with the name Urachal Carcinoma Case Study.ipynb. LC–MS/MS, liquid chromatography with tandem mass spectrometry.

In our previous proteomic study of a chemorefractory metastatic case of urachal carcinoma, we proposed lysine‐specific histone demethylase 1 (LSD1/KDM1A) as a possible druggable target^2^. Here, we extended that study with a broadened default analysis, supplemented with Jupyter notebooks that implement repurposing based on prior knowledge in a reusable pipeline that can be applied to other studies (Fig. 5). Comparing lung tumor to non-cancerous-appearing tissue revealed hundreds of significantly regulated proteins; thus, a strategy for knowledge-derived prioritization, such as text mining and disease and drug associations, became necessary^51,53,54^. The CKG mined the graph to identify drug–target–disease triplets co-mentioned in the literature (3.3 million publications mentioning triplets); to enumerate side effects associated with drugs (72,000 associations); to find similar drugs based on side effects, indications and targets; and to connect drugs with functional pathways.

Among 328 differentially regulated proteins, 188 were upregulated (paired *t*-test; Supplementary Table 2), and 69 had a known association to lung cancer. The CKG not only automatically connected LSD1/KDM1A to tranylcypromine, the drug approved by the tumor board for our patient, but also indicated *trans*-2-phenylcyclopropylamine, a known potent inhibitor of the demethylase, as another treatment option^55^. We identified 60 potential drugs targeting 19 of the prioritized proteins. After retrieving reported side effects associated with the chemotherapeutic regimens used and the identified inhibitors, the CKG re-ranked the remaining drugs according to dissimilar side effects (Jaccard index). A cutoff of less than 0.2 resulted in six drugs (cholic acid, dATP, resveratrol, calcitriol, vorinostat and trichostatin A) targeting six proteins (HDAC1, THBS1, CDH1, CDH17, PTGES and TXN).

Additionally, the CKG suggested publications that co-mention these drugs with their protein targets and the disease and affected tissue, which highlighted the combination of vorinostat and trichostatin A in more than 30 publications. These drugs inhibit HDAC1, a histone deacetylase that induces epigenetic repression linked to tumor progression. Combinations of such inhibitors can inhibit epigenetic silencing and its malignant effects^56–58^. Primed by the involvement of both HDAC1 and LSD1/KDM1A in histone modifications, we extended the analysis to find possible connections between these proteins, which revealed that they are subunits of the CoREST complex, which has recently attracted therapeutic interest and for which the CKG retrieved a paper describing the inhibition of both HDAC1 and LSD1 (ref. ^59^).

### Case studies exemplifiying the capabilities of CKG in sharable notebooks

Our reports directory (Supplementary Fig. 6) includes the sequence of analyses to reproduce the NAFLD and the urachal carcinoma studies described above as well as reanalysis of four additional datasets exemplifying the CKG’s functionality in different contexts. First, we reanalyzed our proteomics investigation of the differences between brown and white adipocytes^60^, prioritizing and annotating significantly regulated proteins known to be associated with metabolic diseases. The resulting knowledge subgraph highlights several inflammation processes and connections to amyloidosis that had previously escaped our attention (Supplementary Fig. 7). Next, the CKG analyzed three studies external to our group, starting with a longitudinal COVID-19 Olink dataset where we reproduced the results comparing COVID-19-positive and COVID-19-negative^61^ and also investigated proteomic differences between the severity groups within the cohort. This analysis highlighted upregulation in proteins such as IL-6, IL-17C, CXCL10 and CCL7, among others, that generally increased with severity and shows that CKG is not limited to MS-based proteomics data. A medulloblastoma multi-level proteomics dataset reanalyzed a tandem mass tag proteomics dataset and employed similarity network fusion to integrate proteomics, PTMs and RNA sequencing data to reveal medulloblastoma subgroups as well as the features that drive these subgroups (Supplementary Table 5)^62^. Finally, in our reanalysis of the CPTAC glioblastoma discovery study (https://cptac-data-portal.georgetown.edu/study-summary/S048), we used the knowledge graph to explore possible drug inhibitors for the list of significantly upregulated proteins found when comparing tumor and normal brain tissue (Supplementary Table 6). This exploratory analysis connected many of the upregulated proteins in tumors to pirfenidone, a drug generally used for idiopathic pulmonary fibrosis known to inhibit TGF-β signaling, a pathway de-regulated in malignant gliomas, and also to reduce the tumor extracellular matrix^63,64^. All these notebooks are part of the CKG’s code repository and documentation and can be visualized at https://CKG.readthedocs.io/en/latest/advanced_features/ckg-notebooks.html.

## Discussion

The CKG represents prior knowledge, experimental data and de-identified clinical patient information in a large network. It harmonizes proteomics data with all this information using a graph structure that naturally provides immediate connections to the identified proteins. We found that its automated, instantaneous and iterative nature helps in revealing pertinent biological context for better understanding and generation of new hypotheses. Furthermore, the graph structure provides a flexible data model that is easily extendable to new nodes and relationships. Although the CKG was particularly designed for answering clinically relevant questions, it is equally applicable to other organisms and to any biological study^65^.

The CKG’s analytics core has an open modular design fully implemented in Python, exploiting open-source libraries that are widely employed and well maintained and that cover a broad data science ecosystem: statistics, network analysis, machine learning and visualization. Using these libraries ensures the quality, robustness and efficiency of the underlying algorithms and methods. It also enables incorporation of new developments in data science, which can quickly be adapted to specifically support proteomics data analysis. Increasing concerns about reproducibility of scientific results^66,67^ are also addressed by the CKG. We employ Jupyter notebooks to generate shareable analysis pipelines that make results reproducible and replicable and envision widespread adoption of this framework by the proteomic community and beyond.

To stimulate the adoption of the CKG, we integrated community-developed standards, such as mzTab and the Sample and Data Relationship Format (SDRF)^68^, for metadata, as well as multiple proteomics data formats generated by commercial or open-source software. Furthermore, the CKG’s modular design facilitates changing these formats and the incorporation of new ones. This flexibility ensures that the CKG will be able to follow an ever-expanding and active MS-based proteomics community as well as other omics initiatives.

**Fig. 6:**
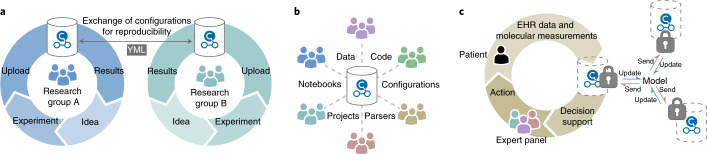
Vision of CKG’s deployment. **a**, Reports and notebooks in local graphs can readily be shared to replicate analyses, thereby contributing to reproducible science. **b**, Aggregating data and knowledge of multiple projects from different groups within a community would allow direct and deep project comparison and lead to increasingly more robust and powerful analysis and knowledge generation. **c**, To protect the sensitive nature of healthcare data and still allow researchers to train models and learn from the data, the CKG could be implemented as a protected graph using federated learning. EHR, electronic health record.

The different components of the CKG allow individual research groups to analyze, integrate and build a database of their proteomics and other omics projects. Reports and notebooks can readily be shared to replicate the analyses, thereby contributing to reproducible science (Fig. 6a). Beyond this, the open nature and free availability of the CKG could allow the aggregation of data and knowledge in what we term a community graph (Fig. 6b). This would ensure that the community benefits from similar proteomics or omics projects performed elsewhere. For biomarker discovery, this constitutes an extension of the ‘rectangular strategy’^69^, allowing direct and deep project comparison and leading to increasingly more robust and powerful analysis and knowledge generation.

We envision that different groups and institutions will have their own local version of the CKG, protecting the sensitive nature of healthcare data, but in a way that still enables cross-platform analyses. New approaches, such as differential privacy and federated learning^70,71^, would allow researchers to use the CKG to train models iteratively across institutions without direct access to the sensitive data (Fig. 6c). Artificial intelligence is set to play an increasing role in MS-based proteomics and biomarker discovery^72^, and we look forward to integrating the CKG with these capabilities as well as taking advantage of novel graph deep learning capabilities.

In conclusion, we describe the CKG, an open, robust framework for transparent, automated and integrated analysis of proteomics and multi-level omics data, designed to incorporate all the prerequisites for reproducible science. The CKG thus directly addresses some of the major bottlenecks toward personalized medicine and the rigorous, data-driven, clinical decision-making process. We expect that others in the biomedical and clinical research community will be encouraged to contribute to and further develop this platform.

## Methods

### Graph database

Graph databases are NoSQL databases that represent and store data using graph structures. The graph structure is a collection of nodes and edges that represent relationships between the nodes and properties. Storage of data in such a structure facilitates access to densely connected data by providing graph traversal linear times. The CKG implements a graph database that contains close to 20 million nodes (36 labels) and more than 200 million relationships (47 different types). The database is built using Neo4j Community Edition (https://neo4j.com/), a scalable native graph database that allows storage, management and analysis of interconnected data. Neo4j provides a query language specific for graph structures, Cypher, and an extensive library of procedures and functions (APOC library and the Data Science library) that can be used for data integration, data conversion or graph analysis. Furthermore, Neo4j makes the database available via several protocols (bolt, http or https) and provides a mission control center that interfaces with the database and helps manage it.

### Data integration

#### Ontologies

To build the CKG database, we selected the different node labels (36 labels) and relationship types (47 types) between them to design the graph data model (Fig. 1c). These nodes and relationships were defined based on the type of biological or clinical questions or the problems set out to respond to or solve. For each node label, we defined the identifiers by using commonly used biomedical ontologies or standard terminologies. Ontologies denote concepts, in this case nodes (for example, diseases), and provide an acyclic graph structure that describes how these concepts are related. We benefited from this underlying structure to integrate these concepts and relationships (‘is_a’ relationships) directly into the knowledge graph. Likewise, we integrated the terms and relationships standardized in terminologies such as SNOMED-CT, which defines clinical terms and their associative relationships.

Some of the nodes in our graph data model could not be described using ontologies or existing terminologies, and they needed to be standardized using identifiers from the selected biomedical databases (for example, UniProt for proteins, HMDB for metabolites and DrugBank for drugs) (Supplementary Table 1).

During the update of the knowledge graph database (graphdb_builder), the reference ontologies, terminologies and databases are updated first and generate dynamically mapping files that are used to standardize the rest of the data. These mapping files are basically dictionaries built using external references (xref attributes) or synonyms provided in the reference ontologies and databases. This system automatically standardizes the different data sources and facillitates updates.

#### Databases

Once the graph data model and the node label identifiers were defined, we selected multiple well-known and used biomedical databases (25 databases) (Supplementary Table 1) to feed the CKG. The selection of databases to be integrated responded to the type of nodes and relationships in the model and was also based on criteria such as access, usability, stability and acceptance by the research community. However, the flexible design of the graph database and the CKG platform allows quick integration of new databases, ontologies, terminologies or even modifications in the original data model (new nodes or relationships) (see Methods, ‘graphdb_builder’ section).

We purposely built in some redundancy by including biomedical databases (for example, DISEASES^73^ and DisGeNET^74^) that provide the same type of relationships, which we used to assess overlap and disagreement of sources (Supplementary Fig. 3b).

#### Experiments

The CKG database models multiple node types, which, in principle, allows integration of different data types: genomics, transcriptomics, proteomics or metabolomics. However, the focus of the graph is initially the integration of quantitative MS-based proteomics data. This might have influenced the structure of the data model specifically on how experimental projects are defined and stored. Similarly, the clinical context in which the database was built limits the data to human, whereas other species are not covered by the graph yet.

Proteomics data can be integrated by creating a new project, which requires defining new nodes in the database: enrolled individuals, biological samples collected from these individuals and analytical samples extracted from those biological samples. Analytical samples correspond to the actual sample analyzed in the mass spectrometer. All these nodes will have external identifiers, and they will be mapped to unique internal identifiers in the knowledge graph. Internal identifiers will then be used to integrate experimental and clinical data seamlessly.

The relationship between analyzed samples and proteins ((Analytical_sample)-[:HAS_QUANTIFIED]-(protein)) will have the quantification (that is, label-free quantification (LFQ) intensity) stored as a property/attribute of the relationship (value). Currently, MaxQuant, Spectronaut, FragPipe, DIA-NN output files and mzTab format or tabular files can be automatically loaded into the database using a specific configuration (YAML file) for each format.

Similarly, clinical data—clinical variables collected for each individual or biological sample (in case of longitudinal studies or multi-specimen studies)—can also be automatically loaded into the database using SDRF^68^ or in tabular format. When the data are provided in tabular format, all clinical variables need to follow the SNOMED-CT standard.

### CKG platform

#### Software architecture

The CKG platform was designed using a modular architecture that divides the platform into functional compartments: graphdb_connector, graphdb_builder, report_manager and analytics_core (Fig. 1a). Each module can be used independently, which provides a flexible environment to cover different scenarios and different needs: direct programmatic interaction with the database, deployment of a local knowledge graph database, visualization of automatically analyzed data from the database or just data analysis and visualization through Jupyter notebooks.

In combination, all modules provide a full workflow from project ideation and creation to analysis and visualization of results (Supplementary Fig. 2). Additionally, we included Jupyter notebooks as another layer of functionality, which allow further and specific analyses and serve as a playground for continuous improvement of the analysis and visualization functionality. Furthermore, notebooks will support replicability, reproducibility and reusability of analysis in the CKG.

All modules were developed in Python 3.7.9. Some of the analyses are performed using R packages (for example, SAMR and WGCNA) called from Python using the Rpy2 library. The library version used in the CKG (rpy2 == 3.0.5) is not compatible with Windows, and these analyses are not available in installations on this operating system. Alternatively, we created a Dockerfile, which holds all the necessary instructions to generate a complete container with all the requirements. In this setup, Windows users have all analyses available. When running the Docker container, four ports will be available: (1) Neo4j HTTP port (7474); (2) Neo4j bolt port (7687); (3) CKG Dash server (8050); and (4) JupyterHub server (8090) (Supplementary Fig. 8). The entry point to the container (docker_entrypoint.sh) defines all the steps needed: start the required services (Neo4j, JupyterHub, redis and celery) and run the report manager dash app. This installation is the easiest and can be used to quickly set up a server version of the CKG with all its components (Python, Neo4j and JupyterHub). Admin users can still customize these services by modifying how the container is built.

All the code can be accessed at https://github.com/MannLabs/CKG, and the documentation is available at https://CKG.readthedocs.io.

##### graphdb_connector

The graphdb_connector provides functionality to connect and query the CKG database. This module is Neo4j dependent. It uses the Python library py2neo, but it is independent from the other functionality in the platform, which allows an agnostic interaction with the database and facilitates adaptation and scalability. Likewise, queries to the database in Cypher language across the platform have been defined as YAML objects with a structure that makes them findable (name, involved nodes and relationships), understandable (description) and easily replaceable.

##### graphdb_builder

This functional module can be used to generate the CKG database. It is divided into two steps: importing and loading. The import (importer.py) downloads the ontologies, terminologies and biomedical databases into the data directory (Supplementary Fig. 6) and formats the data into tabular files (nodes and relationships). The tabular files created by the importer are also stored in the data directory under the Imports folder and organized into ontologies, databases and experiments. Furthermore, the import step generates some statistics (HDF) regarding the number of nodes and relationships formatted as well as file sizes for each ontology, database or experiment. These statistics can be used to track possible errors in the import process (Data/Imports/Stats).

Once the import process finishes, data can be loaded into the graph database by the loader (loader.py), which runs several Cypher queries defined as YAML objects (cypher.yml) and loads the tabular files located in the import folder into the running database. To facilitate this two-step process, we implemented a module called builder (builder.py), which can be used to perform either both steps or one or the other. This module also allows importing or loading of specific ontologies, databases or experiments. After running the two steps, the running database should contain all the nodes and relationships harmonized from the different sources of data.

##### Analytics core

The analytics core is divided into two main functionalities: analytics and visualization. Both modules are independent of the CKG database and can be used to analyze and/or visualize data. The analytics functionality uses Python statistics and Data Science libraries to implement the state-of-the-art analyses of proteomics data (Supplementary Table 2) and incorporates some recent relevant methods, such as WGCNA or Similarity Network Fusion analysis. Moreover, to ensure the correct use of these functions, they are designed to identify the experimental design automatically and, consequently, define the appropriate statistical analysis to perform. The visualization library (viz) uses Plot.ly, an interactive graphing library for Python and R, which opens the possibility to save plots in a format compatible with both programming languages (JSON format).

##### Report manager

The report manager is a tool to interface with the existing projects in the CKG database. This functional module makes use of the analytics core to analyze the project data and generate interactive graphs and then to create detailed reports with these analyses. These reports can be accessed through dashboard apps implemented in Plot.ly Dash (https://plot.ly/dash/). The Dash server can be started by running the index module (index.py) and accessed at http://localhost:5000. The initial app (Home) redirects to the login page, and, once logged in, it shows the current data model and statistics about the database, such as the number of nodes and relationships of each type. Furthermore, this app also links to the other existing pages—Admin, Project Creation, Data Upload and Imports—and lists all the existing projects in the database. The Admin page helps to create new users and update the database by running the importing and loading steps (Supplementary Fig. 9).

When a link to an existing project is accessed for the first time, the report manager runs the automated analyses for each data type in the project using the default configuration. Reports for each data type are shown in tabs in the Project app, and two extra tabs are also present: the multiomics tab, if there is more than one data type (for example, clinical and proteomics data), and the knowledge graph tab, which shows a summarization figure of all the other tabs.

New report pipelines can be defined using configuration files (YAML format) describing the arguments to be used in the data processing, as well as the sequence of analyses to be performed. The structure requires the user, for each analysis’ configuration, to specify which data to use (name of the dataframe(s)), a list of analyses and plots to visualize results (functions in the analytics core: analytics and viz, respectively), whether to store the results as dataframes and the arguments needed for analysis and visualization.

Once generated, project reports are stored in HDF5 format so that they can be quickly shown when accessed again. Project reports can be regenerated either with default configuration or by providing specific configuration files using the ‘Change Analysis’ Configuration’ option in the Project app. The saved reports can also be easily accessed programmatically with functionality within the report manager (project.load_project_report()) or using the R library rhdf5 (see ‘Notebooks’ section).

Reports can be downloaded in a compressed file (zip), which contains one folder for each generated tab, and, inside, all the dataframes created during the analyses (tab separated format, tsv), all the plots as vector and png format and all networks in Graph Modeling Language compatible with Cytoscape and JSON^75^, JSON or the nodes and edges tables.

##### Notebooks

We included Jupyter notebooks as another component of the CKG platform. This component serves three purposes: (1) a playground to test and develop new analyses and visualizations; (2) a collection of recipes that explain how to use CKG’s Python library; and (3) a repository of reanalyses of already published case studies that can be shared, reproduced and reused. The structure of the notebook directory (Supplementary Fig. 6) distinguishes these purposes defining three folders: development, recipes and reports. In the recipes folder, you can find several Jupyter notebooks showing simple functionality and analyses using CKG’s library: how to work with reports in R; how to build a project and generate and visualize a report; how to download and analyze data from PRIDE; how to run power analyses; how to perform batch correction; or how to extract data from the graph database. In the reports directory (Supplementary Fig. 6), we included the sequence of analyses to reproduce the NAFLD and the urachal carcinoma study studies described in the Results.

##### Default analytical pipeline

The initial data preparation step structures the quantified measurements (filtering, imputation, formatting, and normalization), starting with filtering out proteins identified in only a few of the samples (Supplementary Table 2). This filtering step can be specified as a maximum percentage of missing values (default) or as a minimum number of values present per condition (group) or in the entire dataset. For imputation, we implemented several methods that account for missing values of different nature, including the *k*-nearest neighbors (KNN) imputation method, which assumes that the values are missing completely at random (MCAR), and the probabilistic minimum imputation (MinProb) approach for missing values that are considered missing not at random (MNAR) (default)^76^. These two methods can also be combined in a mixed imputation method that considers the percentage of missing values to assume missingness due to MCAR (that is, missingnes <50%) or MNAR otherwise and applies KNN or MinProb, respectively. This step results in a complete matrix called the ‘processed data frame’ and forms the basis for downstream analysis.

Next, we implemented the data exploration step into the workflow to collect summary statistics from the original data (such as number of proteins and peptides). Additionally, it ranks identified proteins according to their average quantified intensity (LFQ^77^) and calculates protein CVs, which can serve as a quality metric.

The subsequent data analysis part includes a dimensionality reduction step and enables visualization of the high-dimensional proteomic datasets using two- or three-dimensional representations. We implemented linear dimensionality reduction (principal component analysis (default)) and nonlinear approaches (*t*-distributed stochastic neighbor embedding (*t*-SNE)) and uniform manifold approximation and projection.

The analytics core enables hypothesis testing, particularly methods for identifying proteins changing significantly between conditions (groups). The default method is ANOVA, but others, such as ANOVA for repeated measurements (ANOVA-rm), *t*-test (independent or paired) or significance analysis of microarrays (SAM), are also available^21^. By default, the analytics core identifies the appropriate test based on the experimental design (for example, independent versus paired and ANOVA versus ANOVA-rm). We also implemented several methods to correct for multiple hypothesis testing, such as Benjamini–Hochberg (BH) FDR (default) or permutation-based FDR, which is used only if the number of permutations specified (default set to 250) is sufficiently large to avoid overestimating false positives.

Strategies for global protein–protein correlation analysis include as default Pearson correlation analysis corrected for multiple testing, which returns a network with identified clusters of correlating proteins (Louvain clustering method). Furthermore, functional enrichment analysis (Gene Ontology and Pathways) enables extraction of potential hypothesis-generating information regarding the functional consequences of proteomics perturbation as an ultimate step in the proteomics analysis (Supplementary Table 2).

##### Machine learning on graphs

The CKG provides functionality to apply machine learning algorithms based on the relationships existing in the knowledge graph. On the one hand, the CKG provides a library of optimized graph algorithms that run within the database framework (using the NetworkX Python library). These algorithms efficiently implement graph analysis tools such as path finding, centrality measurements, community detection and similarity functions, among others. All these algorithms are either directly available in the CKG or through the Graph Data Science library in Neo4j and can be used to effectively identify hidden patterns and generate predictions based on the connected data. Graph-based predictions have been used in multiple scenarios, including drug repurposing, protein–protein interaction (PPI) prediction, disease comorbidity risks or diet-based cancer therapy associations^78–80^. All the types of relationships mined in those studies are part of the CKG and can repeatedly be modeled in the same manner every time new data are integrated. For instance, we used this functionality to map Gene Ontology biological processes to metabolic pathways (Supplementary Table 3). This helps to better interpret functional enrichment results or to connect currently disconnected nodes and extend their annotations—that is, (Biological_processes-[:ASSOCIATED_WITH]-(Metabolite)).

Additionally, application of machine learning algorithms directly on CKG’s graph structure can improve prediction and classification tasks, for instance by using Graph Representation Learning algorithms^37^. To provide an example of the potential of these methods on the CKG’s structure, we used the embedding algorithm Node2Vec (dimensions = 100, walk length = 30, number of walks = 200, *P* = 1, *Q* = 2.0, weight key = score) to represent disease nodes^81^. For that, we first obtained disease-specific subgraphs connecting disease nodes to their associated proteins, modified proteins, metabolites and genomic variants and their relationships (that is, PPIs) from the CKG. We then applied the embedding algorithm to obtain high-dimensional vectors, preserving the properties of these subgraphs for each disease node. When visualizing these embedding representations using *t*-SNE, diseases cluster according to the Disease Ontology anatomical entities that they are annotated to, showing that biological meaning is preserved in these representations (Supplementary Fig. 10). These representations could be used in a variety of machine learning problems, such as node and link prediction, graph classification or graph similarity. When applied to biomedicine, these learning techniques can help stratify patients, build comorbidity networks or repurpose drugs.

### Case studies

#### NAFLD study

We use a previously published internal proteomics dataset^41^ (PXD011839) as a showcase of the capabilities of the CKG. In this publication, Niu et al. studied the plasma proteome profiles of 48 patients with and without cirrhosis or NAFLD and identified several statistically significantly changing proteins, some of which were already linked to liver disease. We aimed to reproduce the results obtained using the automated default analysis pipeline of the CKG.

#### Downstream rapid proteomics analysis

We used a previously published internal proteomics dataset^2^ (PXD008713). This study presents a rapid proteomics analysis that identified a possible alternative treatment for a patient with end-stage cancer. We built a downstream analysis pipeline to accelerate and prioritize alternative candidate drug treatments using the CKG. We provide a Jupyter notebook to show how functionality implemented in the graphdb_connector module (query_utils.py) can be used to single out queries that can help find known links between identified upregulated proteins and inhibitory drugs and between those drugs and known side effects and publications as well as how to use this knowledge to prioritize drug candidates.

#### Multi-level proteomics analysis

We reanalyzed and extended a multi-level proteomics study, including interactomics and phosphoproteomics, that provides insights into the mechanisms of resistance to platinum-based chemotherapy in high-grade ovarian serus adenocarcinoma^3^ (PXD010372). The CKG reproduces the findings and extends them with deeper analysis of the protein complexes identified^82^ and substrate and PhosphoSite-specific annotations^83,84^.

### CKG update

Databases and ontologies integrated in the CKG can be updated using the graphdb_builder. There are two options: full update or partial update. A full update, which will regenerate the entire database with newly downloaded data from the sources, the number of nodes and relationships, will vary from version to version according to changes in these data. On a partial update, the sources to be imported and loaded into the graph need to be specified. The partial update can also be used to extend the graph when a new database or ontology is added. When running a full update, it is recommended to create a different graph database, confirm that the generated graph is correct and then switch to the new database.

Experiments can be updated using the ‘Data Upload’ functionality in the dashboard app by indicating the project identifier and uploading the new data. When a full update is performed in the CKG’s graph, which involves upgrading the version of essential databases, such as UniProt^85^, it is highly recommended to process the raw proteomics data, searching with the new version of the proteome, and to generate again all the project reports with the new data. When this is not possible, we provide a Jupyter notebook to generate a mapping between UniProt versions based on sequence alignment (CKG mapping from fasta.ipynb).

The CKG is an open-source project, and its code will continue to grow and improve through version control in the GitHub repository (https://github.com/MannLabs/CKG). Currently, version 1.0.0 is available, and new releases will be made available in a controlled manner and named following the PEP 440 specification (https://www.python.org/dev/peps/pep-0440/). Because the CKG is an open-source project, contributions can help the framework grow with additional ontology, database or experimental parsers, improved documentation, increased testing and feedback. Specific details on how to contribute can be found in the CKG’s documentation.

### Installation and hardware requirements

The CKG’s purpose and architecture define it as a multi-user platform that requires installation in a server-like setup and with systems administration knowledge. However, individual users can have a local installation, making sure hardware and software requirements are fullfiled. The simplest installation is by using the Docker container and running the ‘minimal’ update in the Admin app (https://ckg.readthedocs.io/en/latest/intro/getting-started-with-docker.html). This installation requires getting access to the licensed databases (SNOMED-CT, DrugBank and PhosphoSitePlus). For specific requirements and installation steps, consult the CKG’s documentation at https://ckg.readthedocs.io/en/latest/intro/getting-started-with-requirements.html.

### Reporting Summary

Further information on research design is available in the Nature Research Reporting Summary linked to this article.

## Online content

Any methods, additional references, Nature Research reporting summaries, source data, extended data, supplementary information, acknowledgements, peer review information; details of author contributions and competing interests; and statements of data and code availability are available at 10.1038/s41587-021-01145-6.

## Supplementary information

Supplementary Information Supplementary Figs. 1–10 and legends for Supplementary Tables 1–6.

Reporting Summary

Supplementary Table Supplementary tables 1-6

## Data Availability

The MS-based proteomics data analyzed in this study were downloaded from the PRIDE database: PXD011839, PXD008713, PXD010372 and PXD008541 (PRIDE database identifiers). Data used in the glioblastoma study were provided by the Clinical Proteomic Tumor Analysis Consortium (NCI/NIH) (https://cptac-data-portal.georgetown.edu/study-summary/S048). The Olink proteomics data were provided by the Massachusetts General Hospital at https://www.olink.com/mgh-covid-study/. A version of our Clinical Knowledge Graph database is at Mendeley Data (https://data.mendeley.com/datasets/mrcf7f4tc2/3) and the Max Planck Institute of Biochemistry (https://datashare.biochem.mpg.de/s/kCW7uKZYTfN8mwg). The databases and ontologies used in the Clinical Knowledge Graph are listed in Supplementary Table 1.

## Code availability

All code is accessible at https://github.com/MannLabs/CKG, and detailed documentation can be found at https://CKG.readthedocs.io.
