# Supplementary material for: A knowledge graph to interpret clinical proteomics data
Source: Nat Biotechnol. 2022 Jan 31;40(5):692–702. doi: 10.1038/s41587-021-01145-6 (PMC9110295; doi:10.1038/s41587-021-01145-6)
Supplement: Supplementary file 1 — Supplementary Figs. 1–10 and legends for Supplementary Tables 1–6. [file 41587_2021_1145_MOESM1_ESM.pdf]

nature portfolio

https://doi.org/10.1038/s41587-021-01145-6

Supplementary information

A knowledge graph to interpret clinical proteomics data

In the format provided by the authors and unedited

nature portfolio

https://doi.org/10.1038/s41587-021-01145-6

Supplementary information

A knowledge graph to interpret clinical proteomics data

In the format provided by the authors and unedited

Supplementary Information -- A Knowledge Graph to interpret Clinical Proteomics Data

Alberto Santos, Ana R. Colaço, Annelaura B. Nielsen, Lili Niu, Maximilian Strauss, Philipp E. Geyer, Fabian Coscia, Nicolai J Wewer Albrechtsen, Filip Mundt, Lars Juhl Jensen, Matthias Mann

Figure S1

a

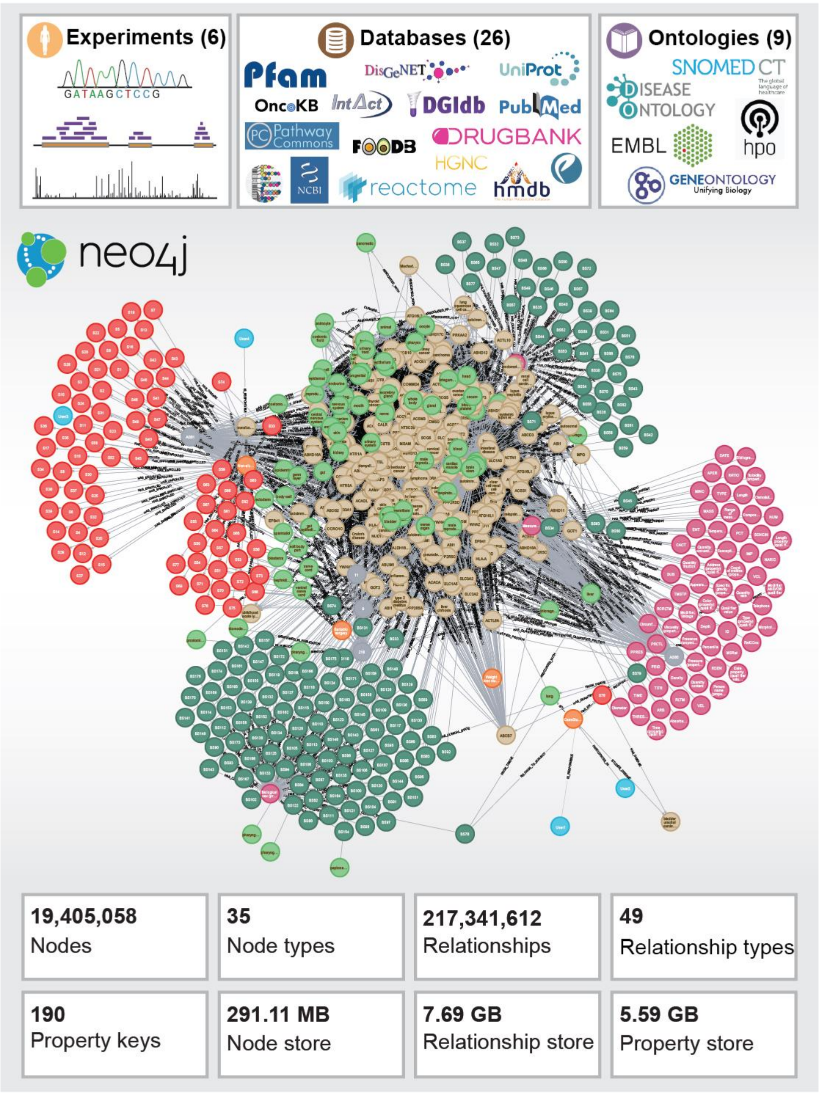

b

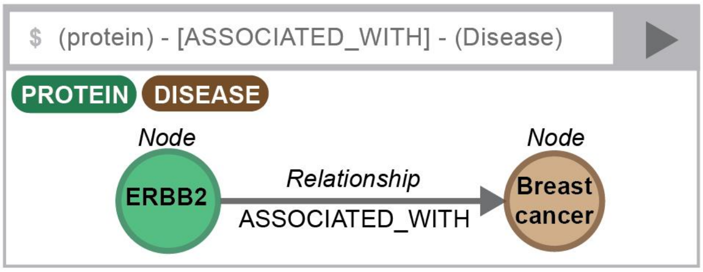

Figure S1. Knowledge Graph Database. a) Snapshot of the current status of the database b) Cypher query language.

Figure S2

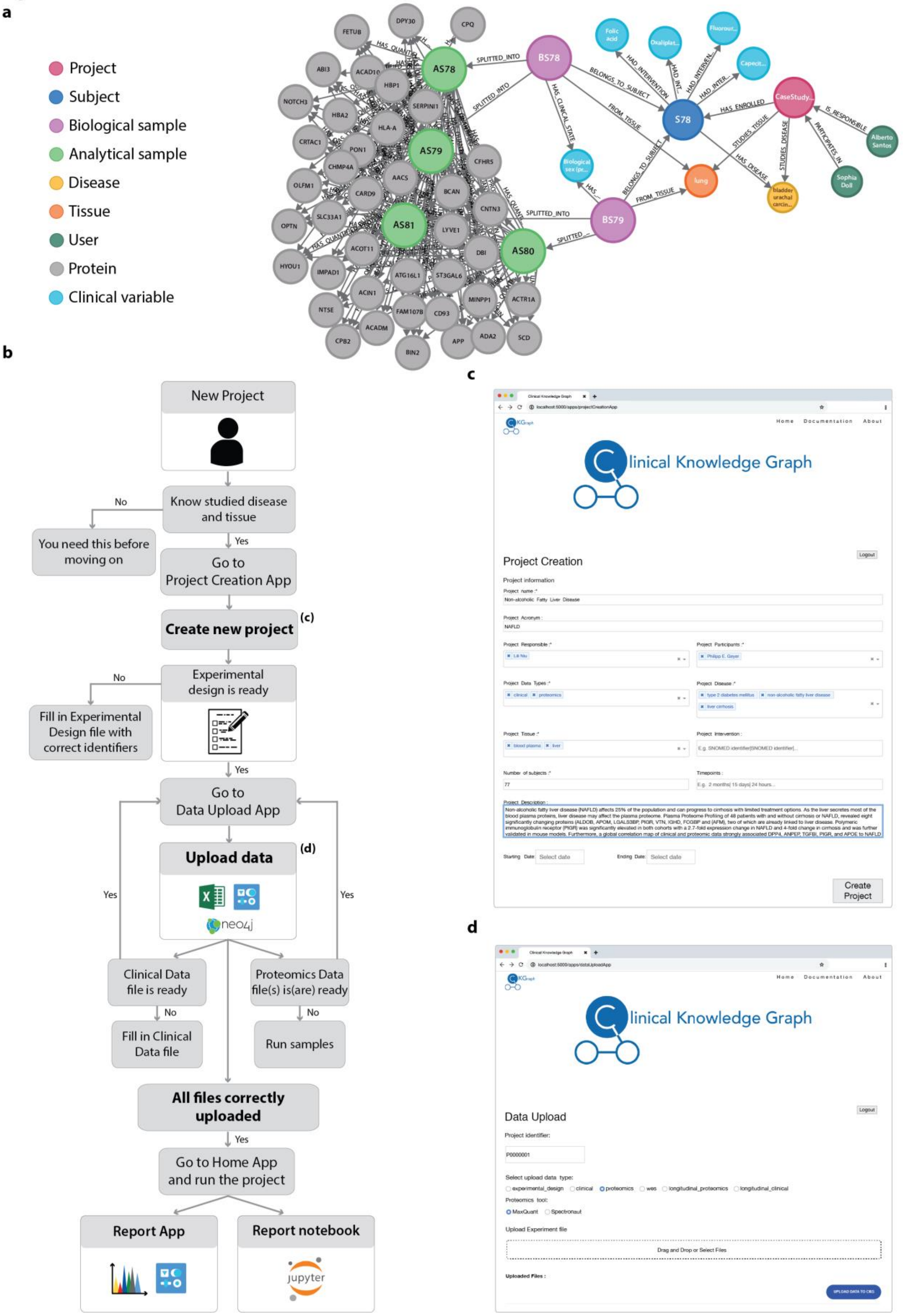

Figure S2. a) CKG Neo4j graph structure showing how metadata is stored around a research project. Nodes for project, subject, biological sample, analytical sample and quantified proteins are depicted,

together with the relationships between them. b) Workflow from project idea to knowledge-based analysis report. Once a project is created, relevant data files can be uploaded, starting with the experimental design file, and followed by the clinical data and proteomics files. When the upload is finished successfully, the user can navigate to the homepage app and run the default analysis pipeline by selecting the respective project. c) Example of how the Project Creation App looks like, and how it should be filled in. d). Data Upload App. Type in the correct project id, select the data type to be uploaded and the appropriate files. Once all files have been selected, press the bottom button to upload the data.

Figure S3

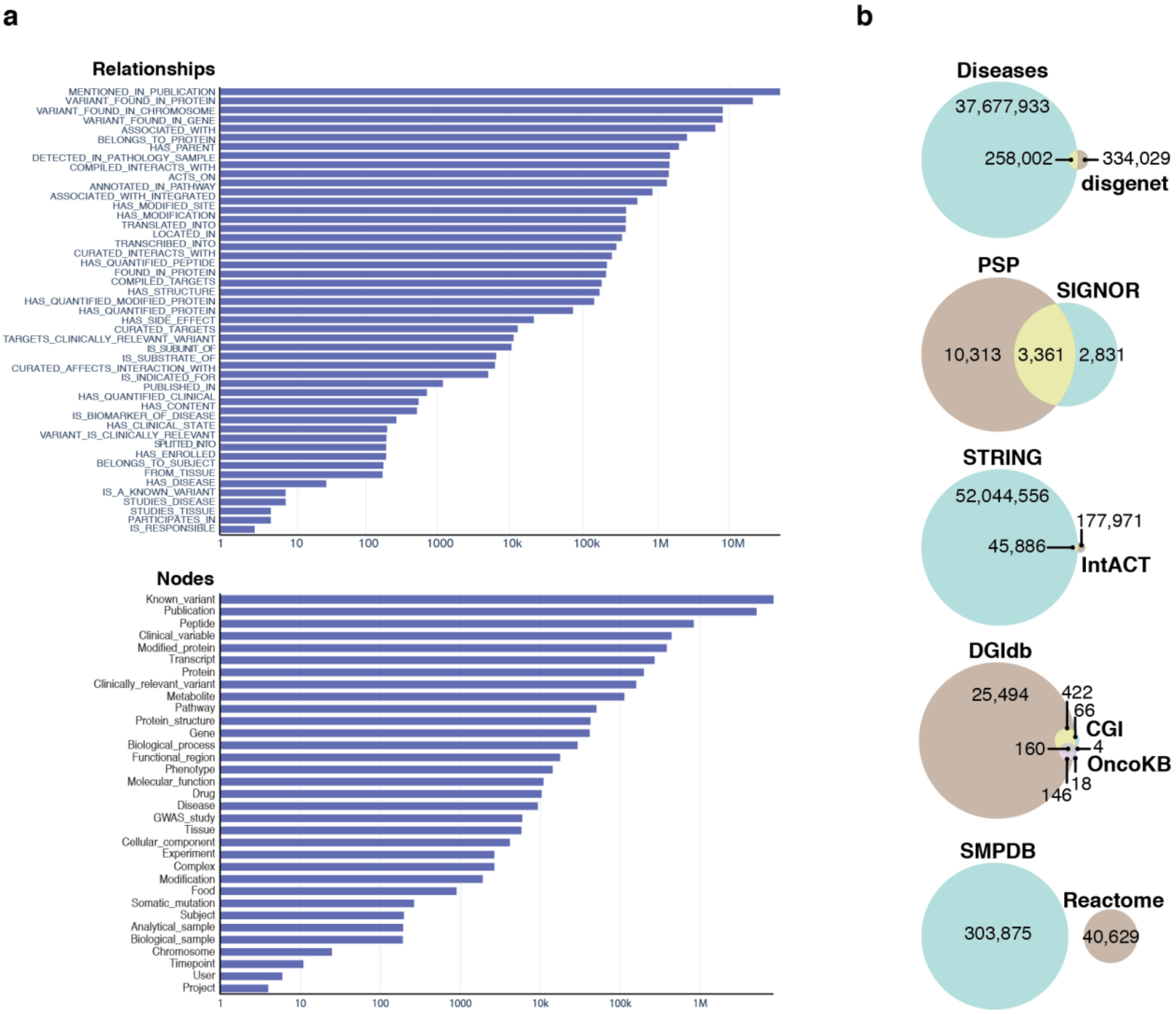

Figure S3. Distributions of nodes, relationships and overlapping sources. a) Barplots of the number of times each relationship- and node type appear in the graph database, respectively. b) Venn diagrams showing the number of relationships originating from different sources, for the five cases where information from multiple databases are used to obtain one relationship type.

Figure S4

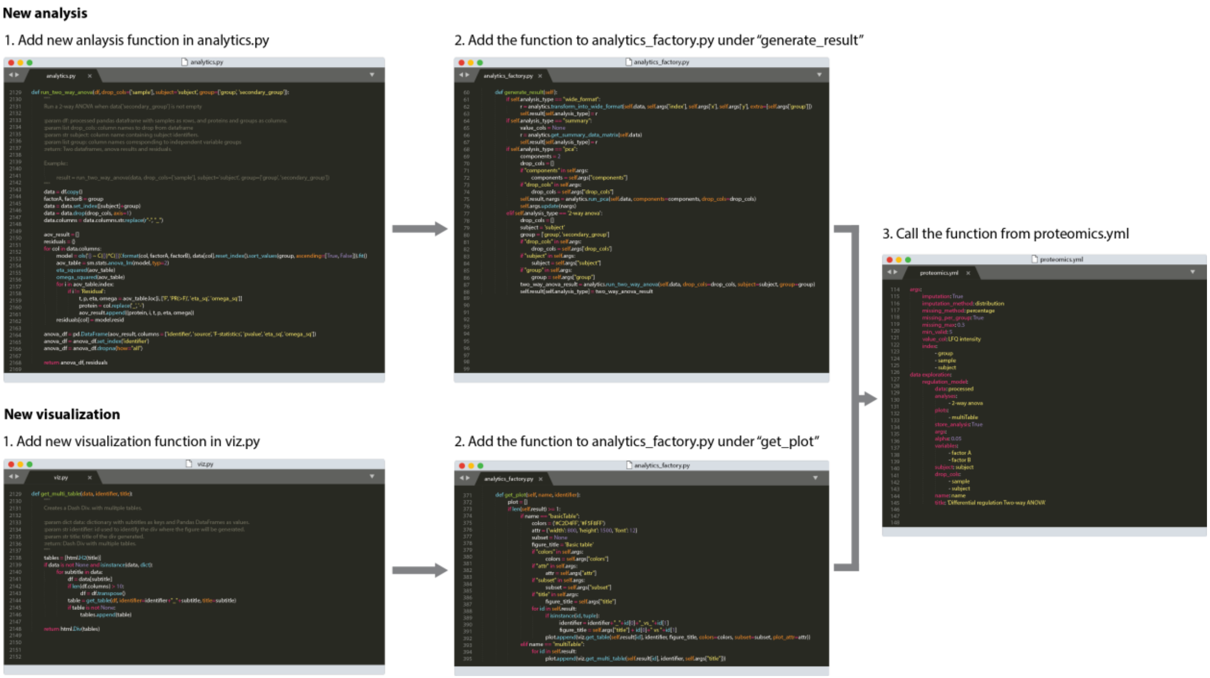

Figure S4. Adding new functions to the CKG. New analysis and/or visualizations can be added to the CKG pipeline in three simple steps: adding the new function to the appropriate *analytics_core* module, adding a call for the function in the *analytics_factory* module and finally, calling it in the appropriate configuration file.

Figure S5

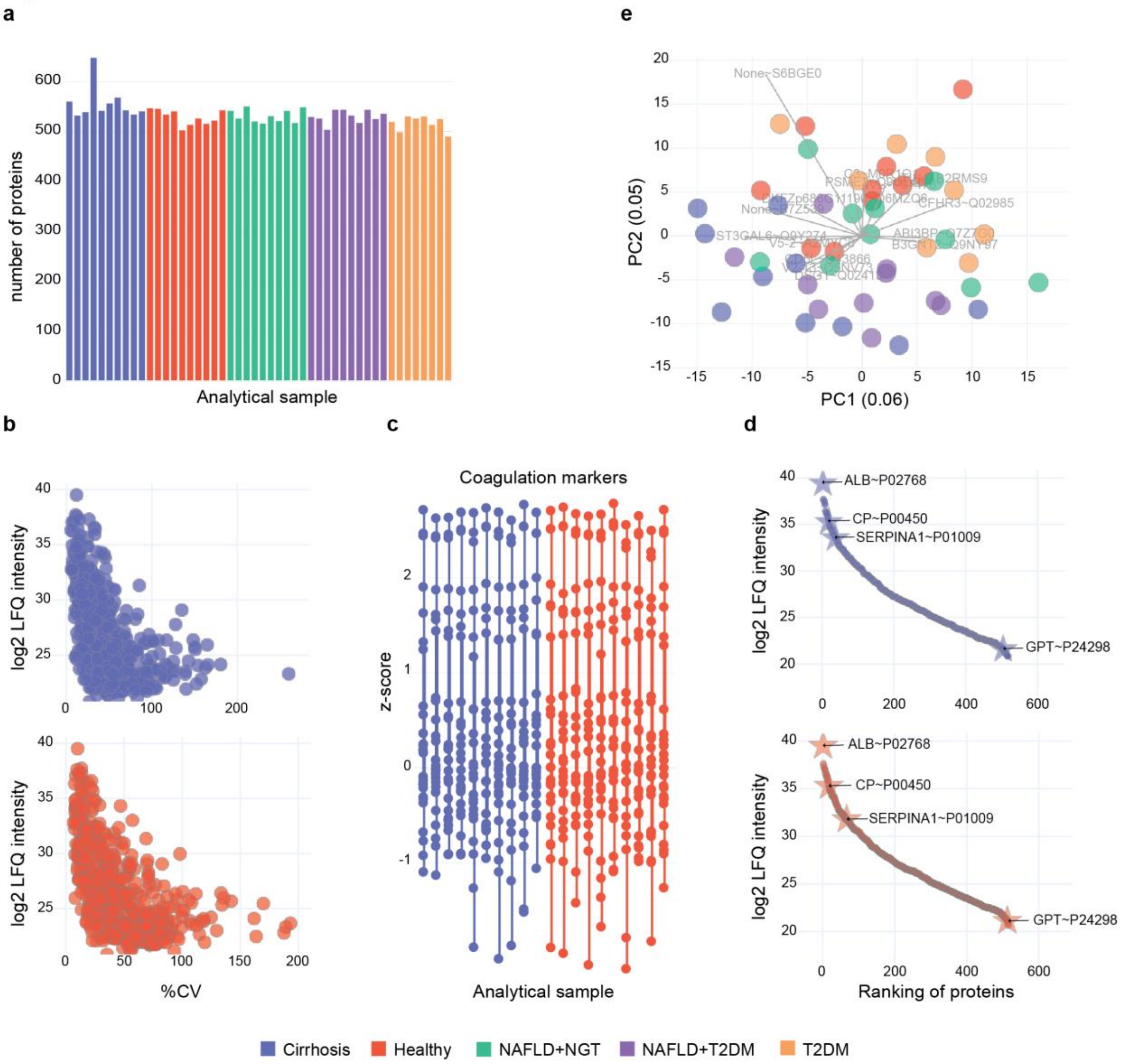

Figure S5. Default analysis pipeline. Additional visualizations generated during the default analysis pipeline. a) barplot showing the number of proteins quantified per sample. b) Coefficient of variation. c) Boxplots showing the distribution of proteins considered quality markers in blood. d) Protein ranking highlighting known clinical markers related to the disease studied (liver-associated diseases). e) PCA plot with top loading proteins and colored by disease group.

Figure S6

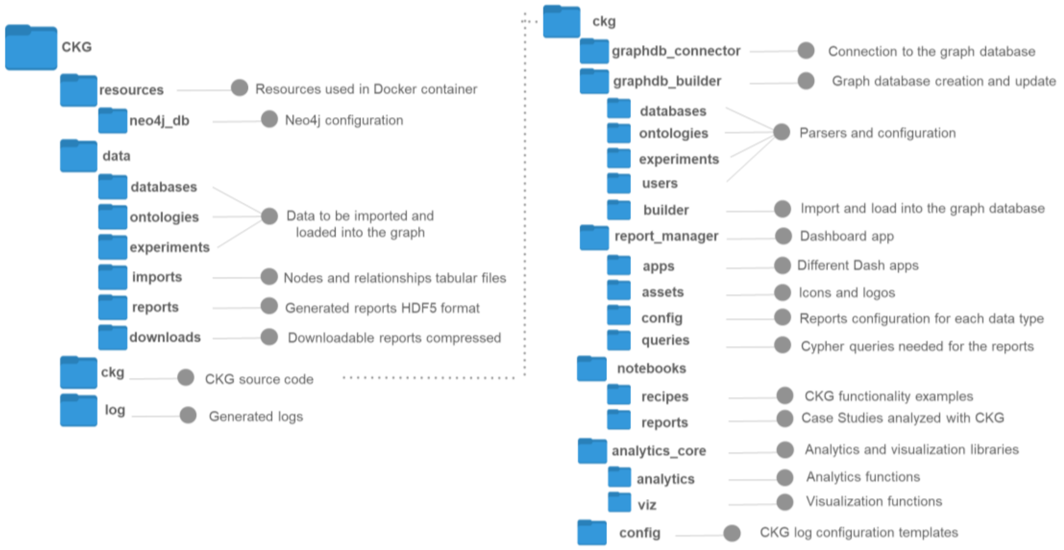

Figure S6. Directory structure. Overview and description of the different directories that form CKG.

Figure S7

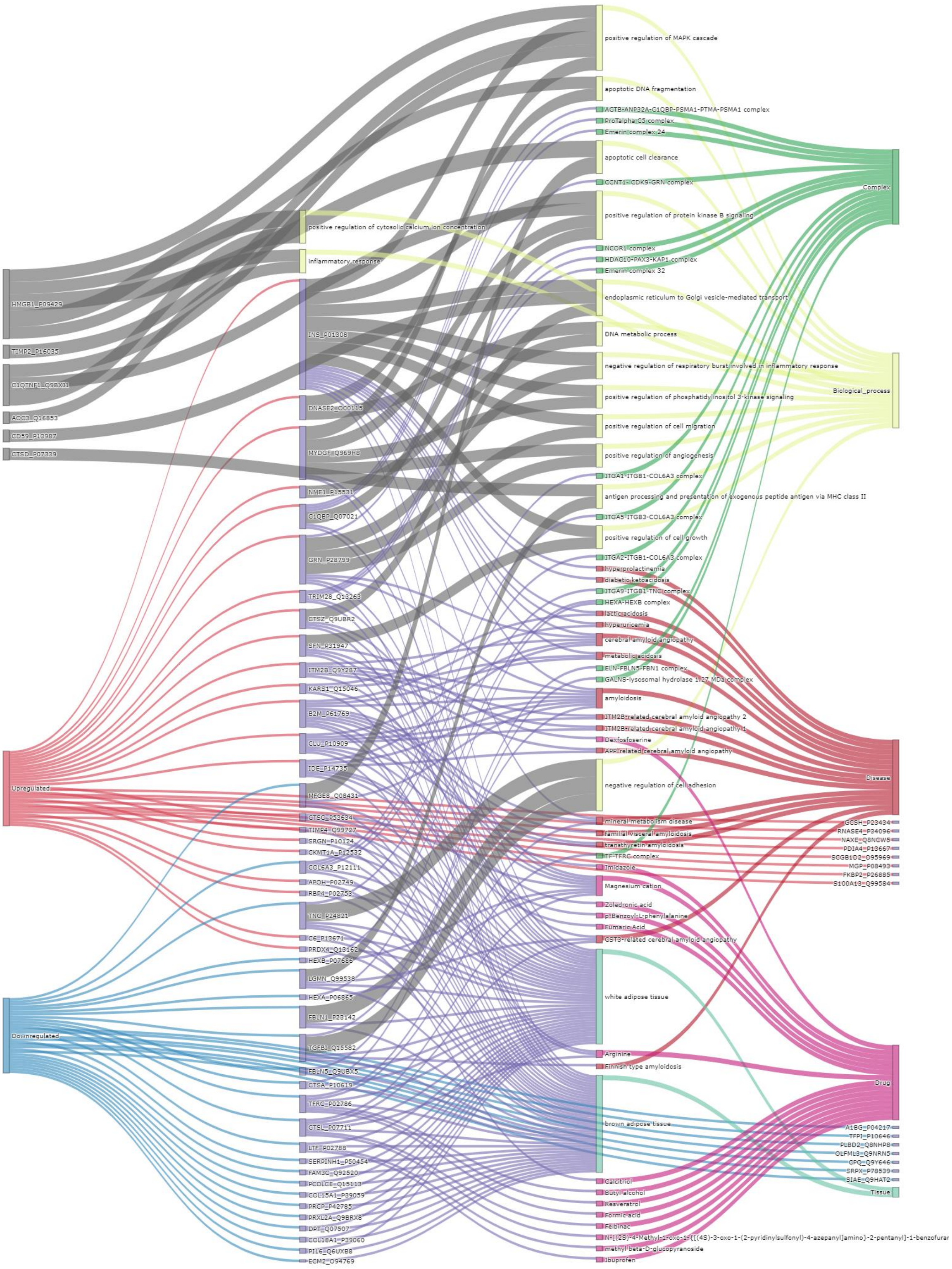

Figure S7. Knowledge Graph Brown vs White fat proteomics. The sankey plot summarizes the knowledge extracted from CKG when annotating secreted proteins differentially regulated when comparing brown and white fat proteomics samples. Data from Deshmukh et al 2019.

Figure S8

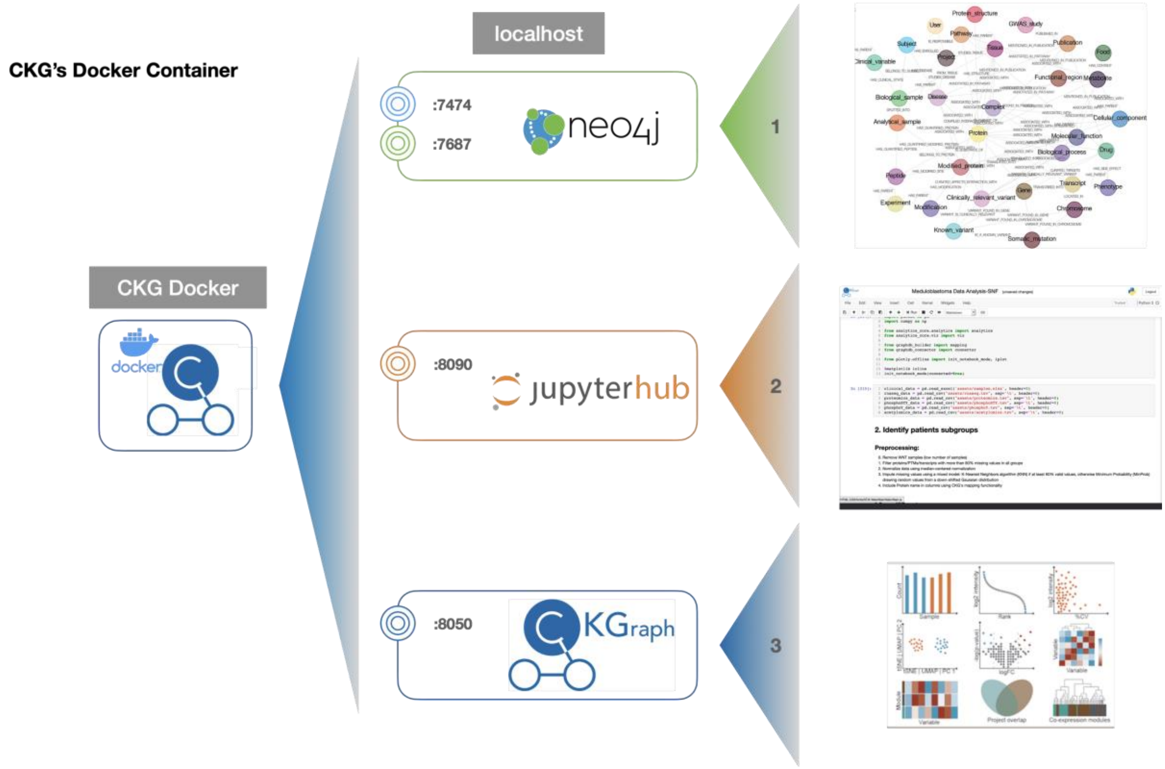

Figure S8. Docker container. CKG can be run through a Docker container built using the Dockerfile provided. This container opens 4 ports that can be mapped locally to access: 1) Knowledge graph database, 2) JupyterHub notebooks and 3) CKG's dashboard app. The instructions on how to build and run the docker container can be found in the documentation (https://ckg.readthedocs.io/en/latest/intro/getting-started-with-docker.html).

Figure S9

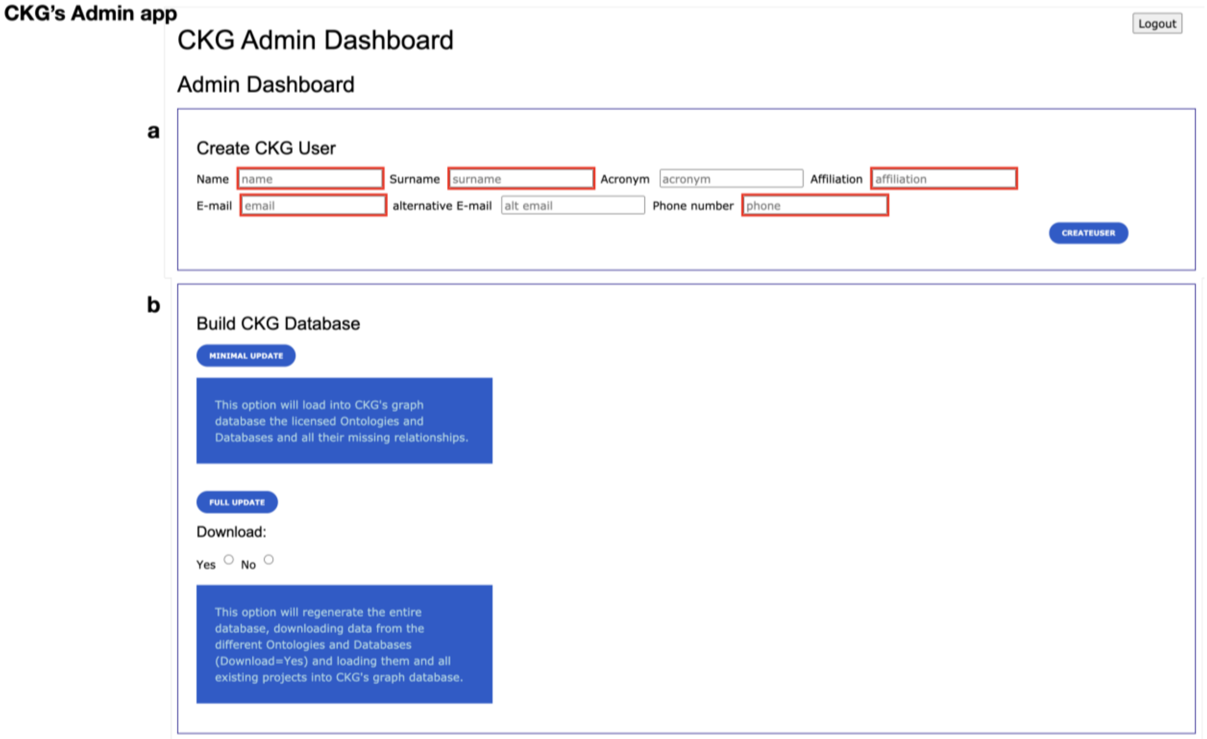

Figure S9. Admin Dashboard. This app is created to provide basic administration functionality: a) creating new CKG users and b) updating the database either with the minimal information (complements the exisiting dump file) with the licenced databases and missing relationships (minimal) or the full update, which downloads all the databases and ontologies and regenerates the entire database.

Figure S10

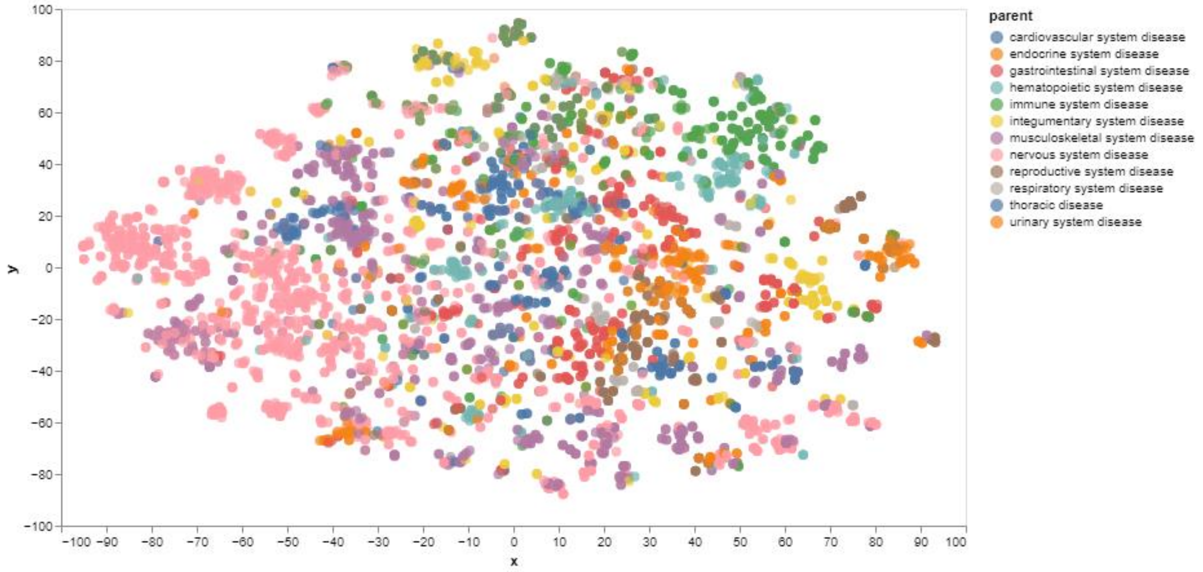

Figure S10. Disease-specific subgraph representation with Node2vec. We use CKG structure to build disease-specific subgraphs connecting associated proteins, protein modifications, metabolites, and variants, and their relationships (i.e. PPIs) and represented them using Node2vec algorithm (dimensions=100, walk length=30, number of walks=200, P=1, Q=2.0, weight key=score). We use the multidimensional generated vectors to visualize disease clusters according to the Disease Ontology anatomical entity they belong to.

Supplementary Tables

Table S1. Databases
List of databases integrated into the Clinical Knowledge Graph database.

Table S2. Analytics core functionality
All the analysis and visualization functions available in the analytics core.

Table S3. Link Prediction
Predicted mapping between Gene Ontology biological processes and Reactome metabolic pathways based on similar protein and metabolite annotations.

Table S4. Protein-drug relationships
Number of protein-drug relationships and the effect of the drugs on these proteins according to STITCH database (http://stitch.embl.de/).

Table S5. Features Prioritized with Similarity Network Fusion
The table summarizes the features driving the identified Medulloblastoma subgroups from each technology (RNAseq, Proteomics, Phosphoproteomics and Acetylomics) from Archer et al 2018.

Table S6. Drug Inhibitors Glioblastoma
List of drug inhibitors connecting proteins upregulated in Glioblastoma when comparing tumor to normal tissue. The data was downloaded from: https://cptac-data-portal.georgetown.edu/study-summary/S048 and were generated by the Clinical Proteomic Tumor Analysis Consortium (NCI/NIH).
