# Supplementary material for: A knowledge graph to interpret clinical proteomics data
Source: Nat Biotechnol. 2022 Jan 31;40(5):692–702. doi: 10.1038/s41587-021-01145-6 (PMC9110295; doi:10.1038/s41587-021-01145-6)
Supplement: Supplementary file 2 — Reporting Summary [file 41587_2021_1145_MOESM2_ESM.pdf]

nature research

Corresponding author(s): Alberto Santos and Matthias Mann

Last updated by author(s): Oct 26, 2021

Reporting Summary

Nature Research wishes to improve the reproducibility of the work that we publish. This form provides structure for consistency and transparency in reporting. For further information on Nature Research policies, see our Editorial Policies and the Editorial Policy Checklist.

Statistics

For all statistical analyses, confirm that the following items are present in the figure legend, table legend, main text, or Methods section.

n/a	Confirmed	
☐	☒	The exact sample size (*n*) for each experimental group/condition, given as a discrete number and unit of measurement
☐	☒	A statement on whether measurements were taken from distinct samples or whether the same sample was measured repeatedly
☐	☒	The statistical test(s) used AND whether they are one- or two-sided<br>*Only common tests should be described solely by name; describe more complex techniques in the Methods section.*
☒	☐	A description of all covariates tested
☐	☒	A description of any assumptions or corrections, such as tests of normality and adjustment for multiple comparisons
☒	☐	A full description of the statistical parameters including central tendency (e.g. means) or other basic estimates (e.g. regression coefficient) AND variation (e.g. standard deviation) or associated estimates of uncertainty (e.g. confidence intervals)
☐	☒	For null hypothesis testing, the test statistic (e.g. *F*, *t*, *r*) with confidence intervals, effect sizes, degrees of freedom and *P* value noted<br>*Give P values as exact values whenever suitable.*
☒	☐	For Bayesian analysis, information on the choice of priors and Markov chain Monte Carlo settings
☒	☐	For hierarchical and complex designs, identification of the appropriate level for tests and full reporting of outcomes
☐	☒	Estimates of effect sizes (e.g. Cohen's *d*, Pearson's *r*), indicating how they were calculated

Our web collection on statistics for biologists contains articles on many of the points above.

Software and code

Policy information about availability of computer code

Data collection	The MS-based proteomics data analyzed in this study were downloaded from the PRIDE database: PRIDE database: PXD011839, PXD008713, PXD010372 and PXD008541 (PRIDE database identifiers). Data used in the Glioblastoma study were provided by the Clinical Proteomic Tumor Analysis Consortium (NCI/NIH): https://cptac-data-portal.georgetown.edu/study-summary/S048<br>The Olink proteomics data were provided by the Massachusetts General Hospital at https://www.olink.com/mgh-covid-study/.<br>The databases and ontologies harmonized in the Clinical Knowledge Graph database were collected using the python libraries in the Clinical Knowledge Graph available at https://github.com/MannLabs/CKG version 1.0 (requires: Python 3.7.9, Neo4j database 4.2.3, R 3.6.1).
Data analysis	All analyses were performed using the Clinical Knowledge Graph available at https://github.com/MannLabs/CKG version 1.0 (requires: Python 3.7.9, Neo4j database 4.2.3, R 3.6.1).

For manuscripts utilizing custom algorithms or software that are central to the research but not yet described in published literature, software must be made available to editors and reviewers. We strongly encourage code deposition in a community repository (e.g. GitHub). See the Nature Research guidelines for submitting code & software for further information.

Data

Policy information about availability of data

All manuscripts must include a data availability statement. This statement should provide the following information, where applicable:

- Accession codes, unique identifiers, or web links for publicly available datasets
- A list of figures that have associated raw data
- A description of any restrictions on data availability

UniProt https://www.uniprot.org/
TISSUES https://tissues.jensenlab.org/

STRING https://string-db.org/
STITCH http://stitch.embl.de/
SMPDB https://smpdb.ca/
SIGNOR https://signor.uniroma2.it/
SIDER http://sideeffects.embl.de/
RefSeq https://www.ncbi.nlm.nih.gov/refseq/
Reactome https://reactome.org/
PhosphoSitePlus https://www.phosphosite.org/
Pfam https://pfam.xfam.org/
OncoKB https://www.oncokb.org/
MutationDs https://www.ebi.ac.uk/intact/resources/datasets#mutationDs
Intact https://www.ebi.ac.uk/intact/
HPA https://www.proteinatlas.org/
HMDB https://hmdb.ca/
HGNC https://www.genenames.org/
GwasCatalog https://www.ebi.ac.uk/gwas/
FooDB https://foodb.ca/
DrugBank https://www.drugbank.ca/
DisGeNET https://www.disgenet.org/
DISEASES https://diseases.jensenlab.org/
DGIdb http://www.dgidb.org/
CORUM https://mips.helmholtz-muenchen.de/corum/
Cancer Genome Interpreter https://www.cancergenomeinterpreter.org/
Disease Ontology https://disease-ontology.org/
Brenda Tissue Ontology https://www.brenda-enzymes.org/ontology.php?ontology_id=3
Experimental Factor Ontology https://www.ebi.ac.uk/efo/
Gene Ontology http://geneontology.org/
Human Phenotype Ontology https://hpo.jax.org/
Units Ontology https://bioportal.bioontology.org/ontologies/UO
SNOMED-CT http://www.snomed.org/
Protein Modification Ontology https://www.ebi.ac.uk/ols/ontologies/mod
Molecular Interactions Ontology https://www.ebi.ac.uk/ols/ontologies/mi
Mass Spectrometry Ontology https://www.ebi.ac.uk/ols/ontologies/ms

The MS-based proteomics data analyzed in this study were downloaded from the PRIDE database: PRIDE database: PXD011839, PXD008713, PXD010372 and PXD008541 (PRIDE database identifiers). Data used in the Glioblastoma study were provided by the Clinical Proteomic Tumor Analysis Consortium (NCI/NIH): https://cptac-data-portal.georgetown.edu/study-summary/S048
The Olink proteomics data were provided by the Massachusetts General Hospital at https://www.olink.com/mgh-covid-study/.
A version of our Clinical Knowledge Graph database at Mendeley Data (https://data.mendeley.com/datasets/mrcf7f4tc2/3) and also at the Max Planck Institute of Biochemistry (MPIB) (https://datashare.biochem.mpg.de/s/kCW7uKZYTfN8mwg)

Field-specific reporting

Please select the one below that is the best fit for your research. If you are not sure, read the appropriate sections before making your selection.

☒ Life sciences ☐ Behavioural & social sciences ☐ Ecological, evolutionary & environmental sciences

For a reference copy of the document with all sections, see nature.com/documents/nr-reporting-summary-flat.pdf

Life sciences study design

All studies must disclose on these points even when the disclosure is negative.

Sample size	This study is a re-analysis of other published studies. No sample size analysis was needed.
Data exclusions	This study is a re-analysis of other published studies. No data were excluded.
Replication	The analysis pipeline is defined in a configuration file and can be easily replicated on a different data set.
Randomization	This study is a re-analysis of other published studies. Randomization is not applicable since we used existing study designs.
Blinding	This study is a re-analysis of other published studies. Blinding is not applicable since we used existing study designs.

Reporting for specific materials, systems and methods

We require information from authors about some types of materials, experimental systems and methods used in many studies. Here, indicate whether each material, system or method listed is relevant to your study. If you are not sure if a list item applies to your research, read the appropriate section before selecting a response.

Materials & experimental systems

n/a	Involved in the study
☒	☐ Antibodies
☒	☐ Eukaryotic cell lines
☒	☐ Palaeontology and archaeology
☒	☐ Animals and other organisms
☒	☐ Human research participants
☒	☐ Clinical data
☒	☐ Dual use research of concern

Methods

n/a	Involved in the study
☒	☐ ChIP-seq
☒	☐ Flow cytometry
☒	☐ MRI-based neuroimaging
